# Biocompatible SWCNT Conductive Composites for Biomedical Applications

**DOI:** 10.3390/nano10122492

**Published:** 2020-12-11

**Authors:** Aleksandr Markov, Roger Wördenweber, Levan Ichkitidze, Alexander Gerasimenko, Ulyana Kurilova, Irina Suetina, Marina Mezentseva, Andreas Offenhäusser, Dmitry Telyshev

**Affiliations:** 1 Institute for Bionic Technologies and Engineering, I. M. Sechenov First Moscow State Medical University, 119991 Moscow, Russia; ichkitidze@bms.zone (L.I.); gerasimekno@bms.zone (A.G.); telyshev@bms.zone (D.T.); 2 Institute of Biological Information Processing, Bioelectronics (IBI-3), Research Center Jülich, 52425 Jülich, Germany; r.woerdenweber@fz-juelich.de (R.W.); a.offenhaeusser@fz-juelich.de (A.O.); 3 Institute of Biomedical Systems, National Research University of Electronic Technology, Zelenograd, 124498 Moscow, Russia; kurilova@bms.zone; 4 Ivanovsky Institute of Virology, N. F. Gamaleya National Center of Epidemiology and Microbiology, 123098 Moscow, Russia; ikas@inbox.ru (I.S.); marmez@mail.ru (M.M.)

**Keywords:** biocompatibility, bovine serum albumin, carboxymethylcellulose, fibroblasts, conductive composites

## Abstract

The efficiency of devices for biomedical applications, including tissue engineering and neuronal stimulation, heavily depends on their biocompatibility and performance level. Therefore, it is important to find adequate materials that meet the necessary requirements such as (i) being intrinsically compatible with biological systems, (ii) providing a sufficient electronic conductivity that promotes efficient signal transduction, (iii) having “soft” mechanical properties comparable to biological structures, and (iv) being degradable in physiological solution. We have developed organic conducting biocompatible single-walled carbon nanotubes (SWCNT) composites based on bovine serum albumin, carboxymethylcellulose, and acrylic polymer and investigated their properties, which are relevant for biomedical applications. This includes ζ-potential measurements, conductivity analyses, and SEM micrographs, the latter providing a local analysis of SWCNT distribution in the base material. We observed the development of the electrical conductivity of the SWCNT composites exposed to 1 mM KCl electrolyte for 40 days, representing a high stability of the samples. The conductivity of samples reaches 1300 S/m for 0.45 wt.% nanotubes. Moreover, we demonstrated the biocompatibility of the composites via cultivating fibroblast cell culture. Finally, we showed that composite coating results in the longer lifespan of cells on the surface. Overall, the SWCNT-based conductive composites might be a promising material for extended biomedical applications.

## 1. Introduction

The development of biocompatible and at the same time partially biodegradable materials with specific properties represents an important issue in biomedical research. In this context, biocompatible conducting materials are of special interest, e.g., for biomedical applications ranging from tissue engineering, drug delivery, and bioimaging, to biosensing [1,2,3,4,5,6,7,8,9,10]. Particularly, tissue engineering with electrical stimulation allows to regulate various cellular behaviors like cellular adhesion, alignment, proliferation, or differentiation, and, thus, facilitate the regeneration of damaged tissues, such as skin, bone, nerve, or myocardium tissues [11,12,13,14,15,16,17].

In the 1980s, conducting polymers became a very promising alternative to conventional metals [18,19] or ceramics [20,21] in this field. With an electrical conductivity approaching those of metals and a good processability, they seemed to represent perfect biocompatible conducting systems [22,23,24]. However, there are practical problems when polymers with suitable conductivity are used in biomedical applications ranging from poor polymer–cell interactions and uncontrollable mechanical properties to even bioincompatibility effects (e.g., inflammatory responses to implanted polymeric biomaterials) [25,26,27,28,29,30]. A possible solution for these limitations represents the combination of intrinsically biocompatible and partially biodegradable material with conducting material. The idea to mix biocompatible material and conductive polymers to create new composite biocompatible conductive materials is not novel. Conductive polymers allow excellent control of the electrical stimulus, possess very good electrical and optical properties, have a high conductivity/weight ratio, and can be made biocompatible and biodegradable [2,31,32]. Moreover, it has been shown that many natural-based polymers like albumin or cellulose and even some synthetic polymers are perfect biodegradable biomaterials [33]. However, these materials lack of ionic and electronic conductivity. Therefore, a combination of nonconducting natural or artificial polymers with a highly conductive material offers new options for biomedical applications requiring conductive biocompatible components [34,35,36].

Currently, new polymers that can increase the cell proliferation and the tissue regeneration are of a high interest. Methods for engineering of these polymers can be divided into the following groups. The first group includes the more traditional methods (electrospinning, phase separation, solvent casting and particulate leaching, melt molding, etc.) [37]. The second group includes modern methods of the 3D prototyping (extrusion, drip printing, stereolithography, selective laser sintering, two-photon polymerization, fused deposition modeling, etc.). Advantages of the modern methods are the high resolution and the ability to precisely control the internal structure of the obtained materials [38]. Biodegradable polymers must have a specific porosity and a rough surface for the better cell attachment. The mechanical characteristics of the polymers must correspond to the type of tissue that the material will interact with or replace. Most commonly used in biomedicine are natural (collagen, chitosan, gelatin, alginate, and silk) and synthetic polymers (polylactic, polyglycolic acid, polycaprolactone, etc.) [39]. The major disadvantages of these polymers are their low mechanical strength and electrical conductivity. As a result, they are used in combination with nanoparticles [40]. These nanoparticles increase the strength of the material, while the conductive nanoparticles give the materials the specific electrical conductivity necessary for the regeneration of conductive tissues, such as the heart and nerves. It was shown that electrically conductive polylactic acid composites with carbon nanotubes can be used for the proliferation of cardiac myoblasts [41]. The SWCNTs enhanced the alignment of polymer chains, which led to an increase in the crystallinity of the composites. At the same time, the composites showed an increase in the elastic modulus and the electrical conductivity by 6–9 orders of magnitude in the direct dependence on the CNT concentration. The results fluorescence imaging demonstrated successful attachment of myoblasts to the composite and their proliferation. Li et al. created conducting nanocomposites made of CNT and sericin, which helped to restore the damaged peripheral nerve in a rat model 12 weeks after the implantation [42]. The nanocomposites had a porous microarchitecture and had electrical conductivity due to the inclusion of carbon nanotubes. Restoration of the peripheral nerve integrity was achieved by connecting the electrodes to the nanocomposite and the subsequent electrical stimulation. This procedure improved the functioning of the restored nerve tissue.

Here, we report on the engineering of biocompatible conductive composites based on the biocompatible polymers bovine serum albumin (albumin) [43], carboxymethylcellulose (cellulose) [44], and poly(methyl methacrylate) (acrylic) [45,46] in combination with single-walled carbon nanotubes (SWCNT). The composites are mixed with different ratios (0.05–0.45% SWCNTs) and deposited as thin films (~5 µm) on inorganic carriers. We demonstrate that the conductivity can be varied in an extremely broad regime ranging from 10^−8^ to 10^3^ S/m. Furthermore, the degradability is analyzed via long term (two weeks) exposure of these composites to an electrolyte (0.1 mM KCl) and determining the resulting degradation of the surface potential and morphology change. In order to reveal a biocompatibility of the samples, we perform a successful cultivating of fibroblast cell culture on the surface of thin SWCNT-based composites films. The fluorescent microscopy of stained live cells shows an increased cell density in comparison to standard values and a positive influence on the lifespan of cells. As a result, we are able to demonstrate that engineered SWCNT combine conductive and biocompatible properties required in a variety of biomedical applications.

## 2. Materials and Methods

### 2.1. Molecules and Organics

Albumin (bovine serum albumin, CAS Number: 9048-46-8) was obtained from AMFESCO (Plainview, New York, NY, USA). The electrokinetic potential (ζ-potential) of unmodified albumin is −(6 ± 0.5) mV [47]. Cellulose (carboxymethylcellulose, viscosity of 6000–6500 cps) was obtained from JRS (Berlin, Germany), which has a ζ-potential of unmodified cellulose of −(37 ± 1) mV [48]. Acrylic (poly(methyl methacrylate)) was obtained from Sigma-Aldrich (Darmstadt, Germany). No ζ-potential value for poly(methyl methacrylate) mixed with water is available in literature. Herein and further the term unmodified shows that the surface of albumin, cellulose or Si/SiO_2_ is not affected by any additional treatment that can modify surface properties, i.e., intensive solvent cleaning, plasma treatment, etc.

### 2.2. Substrates Cleaning

In this study, p-doped (111)-oriented silicon 4-inch wafer (Si-Mat, Landsberg am Lech, Germany, 3.6−6.5 Ω·cm) with a 90 nm thick SiO_2_ termination layer cut into smaller samples with a size of 10 mm × 10 mm was used. All substrates were cleaned in acetone (>99.9%, Sigma-Aldrich, Darmstadt, Germany) in an ultrasonic bath for 5 min (25 °C at 320 W power and 37 kHz frequency) and subsequently in isopropyl alcohol (2-propanol, >99.8%, Sigma-Aldrich, Darmstadt, Germany) in an ultrasonic bath for 5 min using the same parameters as before. The substrates were then dried in nitrogen gas flow. After that substrates were exposed to UV-generated ozone (Ossila UV Ozone Cleaner, Sheffield, UK) for 15 min in order to remove residuals of organic contamination.

### 2.3. Streaming Current Measurements

In order to analyze the surface potential of the samples, a modified electrokinetic analyzer (SurPASS, Anton Paar Germany GmbH, Ostfildern, Germany) was used. A pair of identical planar substrates (10 mm × 10 mm) was placed in a clamping cell, with the surfaces to be analyzed facing each other and forming a microfluidic channel (see Figure S1). To obtain a large signal, a small separation (typically ~70 μm) between the two plane-parallel surfaces was chosen that still enabled a laminar flow of the electrolyte. The ζ potential was determined using the Helmholtz−Smoluchowski equation [49], *ζ =* (*dI/dp*) (*ηL*/*εε_o_A*), where *p* is the pressure necessary to generate the laminar flow; *η* and *ε* are the viscosity and dielectric constant of the electrolyte, respectively; *ε_o_* is the vacuum permittivity; *L* and *A* represent the length and cross-section of the flow channel, respectively; and *I* is the resulting current measured between two electrodes placed at each side of the measuring cell. The resulting ζ potential represents the electrokinetic potential at the shear plane between the mobile and immobile Helmholtz layers and is a measure for the surface potential [49]. The pH value of the electrolyte is adjusted via titration. Electrolyte and titration solutions are aqueous solutions based on Milli-Q water containing potassium chloride (1 mM KCl) in the case of the working electrolyte and a gradual titration using a basic electrolyte of 50 mM KOH. During the experiment, the temperature of the medium was kept constant at 25 °C. Furthermore, the reservoirs for the working electrolyte and the titration electrolyte were encapsulated to minimize contact with air. A N_2_ purger was used to suppress the formation of hydrocarbonates (HCO_3_^−^), which can form if CO_2_ is dissolved in the aqueous solution. Prior to each experiment, the experimental setup was rinsed with 2 L of Milli-Q water and subsequently with the working electrolyte solution. Before each series of measurements, pH and conductivity electrodes were calibrated with the corresponding calibration buffer solutions [50].

### 2.4. Fibroblast Cell Culture

Human fibroblast cell line (FH-T), which was acquired at the cell culture collection of the National Research Center for Epidemiology and Microbiology of the Ministry of Health of the Russian Federation, was used. Cells were cultured in Dulbecco’s Modified Eagle Medium (DMEM)–90% culture medium supplemented with 10% calf fetal serum in a 6-well plate. Immediately prior to the incubation, samples were rinsed in DMEM for 10 min to eliminate contaminants. Determination of the exact number of cells taken in the experiment was carried out immediately before seeding the freshly prepared culture mixture in the wells with the sample using an automatic cell counter (Scepter Millipore, Merck KGaA, Darmstadt, Germany). The FH-T cell concentration was approximately ≈4 × 10^4^ cells/mL. Cells were incubated for 24 and 48 h in a thermostat with 5% CO_2_ at 37 °C.

The samples for the cell line were prepared under sterile conditions. Directly after preparation, they were irradiated with ultraviolet light for 20 min. Further, they were first washed with a cell-free medium. Samples were placed on the bottom of the wells, a few milliliter of the medium was poured into each well with the sample, kept for a few seconds, then the medium was removed. Finally, the medium with cells was filled in.

### 2.5. Cell Imaging

Live cell staining was performed using 10 mg/mL Hoechst 33,342 and 5 μg/mL ethidium bromide (both Life Technologies, New York, NY, USA) in a supplemented cell growth medium to stain body and nucleus of cells in green and red, respectively. Cells with Hoechst 33,342 were incubated for 15 min at 37 °C, and then with ethidium bromide for 5 min at 37 °C. The live imaging was conducted at day in vitro (DIV) 1 and DIV 2. The fluorescence imaging was performed via a laser scanning microscope (LSM880, Carl Zeiss, Berlin, Germany) using the Zen software (Zen 2.3 SP 1 FP3 Black, Carl Zeiss Microscopy GmbH, Jena, Germany) (see Figure S2). In each case, three images were acquired. The SEM images were obtained on a NVision 40 SEM (Zeiss, Oberkochen, Germany).

### 2.6. Preparation of Composites

SWCNT were synthesized by the electric arc method on a Ni/Y catalyst and subsequently purified by rinsing with a dispersion of HNO_3_/H_2_SO_4_ until neutral reaction. The average diameter and length of the nanotubes are approximately 1.4–1.8 nm and 0.3–0.8 μm, respectively. The resulting specific surface area is ≈400 m^2^/g and the purity of the SWCNT is 98%. However, SWCNT are connected into bundles under the Van der Waals force and their average dimensions significantly increase. In order to create a mixture of nanotubes with any material with desirable wt%, following steps were performed.

(i) An aqueous dispersion (see Table S1) for the later sonication was produced by adding the appropriate amount of nanotubes to Milli-Q water.
(ii) An ultrasonic homogenizer (Sonicator Qsonica, Newtown, CT, USA, with maximum power of 700 W) was used in order to homogeneously distribute nanotubes in the water. The sonication was performed at 35–40% of the maximum power for 60 min. In order to avoid overheating during the procedure, the glass beaker with water and nanotubes was cooled by ice water.
(iii) Right after the sonication, the obtained dispersion was mixed with albumin, cellulose, or acrylic polymer, respectively. The resulting compound was thoroughly mixed using a magnetic stirrer for 4 h.
(iv) Afterwards, the dispersion was decanted within 24 h, and then mixed again via a magnetic stirrer for 30 min.
(v) Finally, the composite material is sonicated for 30 min right before the drop casting via the laboratory pipette on the cleaned Si/SiO_2_ substrates.

Directly after the Si/SiO_2_ sample cleaning, a freshly prepared composite is distributed on the surface of the sample via the spin coater at a relatively low speed of 100 RPM. Out of experiments samples are kept in the sterile container.

An example of single-walled carbon nanotubes layer prepared from the water dispersion without composite material is shown in Figure S3. It should be noted that the nanotubes introduced a significant change in the morphology of albumin-based composite. In its virgin state (without nanotubes) deposited albumin films dry with the appearance of cracks all over the surface (see Figure S4), whereas an addition of nanotubes (even a small amount (0.05 wt.%)) reduces the strain in the film and prevents it from cracking.

### 2.7. Electrical and Thickness Measurements

We analyzed the sample’s resistance via four probe measurement technique using a multimeter (Keithley 2000, RS Components Ltd., Corby, UK). The thickness of the samples was measured via the surface profilometry (Dektak 150, Veeco, New York, NY, USA).

## 3. Results and Discussion

For the analysis of the biodegradability and conductive behavior of the different composites, polymers with SWCNT have been deposited via drop-casting onto planar Si/SiO_2_ substrates (see Materials and Methods section). The stability, which correlates to the biodegradability, and the conductance of the resulting films with a thickness of 5 µm have been tested via surface potential analysis and resistive four probe measurements. We explicitly chose surface potential analyses since (i) the surface of the films exposed to an electrolyte provides the best measure for any degradation of the film and (ii) since it is known that the surface potential plays an important role for a cell adhesion and proliferation [51,52]. Although these experiments (surface potential and resistance) represent mean-field methods and, thus, provide integral properties of the layer, they are well established for the characterization of the development of a surface. Furthermore, the analyses are supported by scanning electron microscopy (SEM) and fluorescence microscopy measurements. Let us start with the results obtained for the surface potential measurements.

### 3.1. Surface Potential

Streaming current measurements (see **B** section) are performed to analyze the electrokinetic potential (ζ-potential) of the three different composites using the films with the highest concentration of nanotubes, i.e., composites with 0.45 wt% SWCNT. Each sample is measured 3 times for a duration of 6 h in a streaming electrolyte of 1 mM KCl at a pH of 5.5 ± 0.2. Between these measurements, the sample is stored for 1 week in an identical electrolyte (1 mM KCl at pH 5.5). This allowed us to record the stability of the layer over the time of ≈15 days.

**Figure 1:**
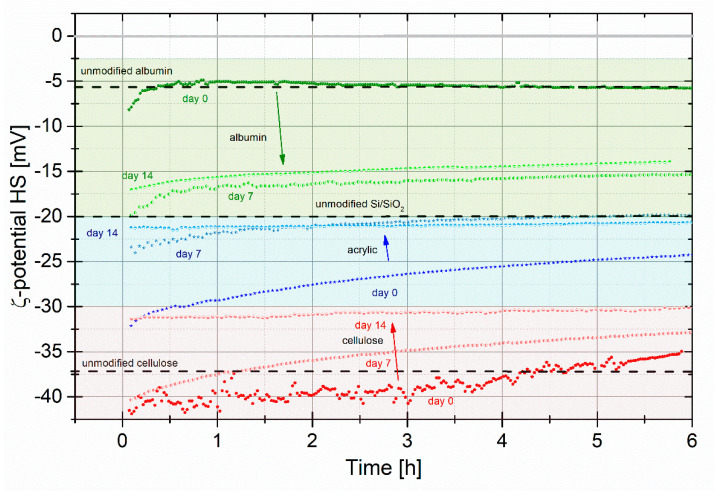
ζ-potential stability tests (measured in 1 mM KCl at a pH of 5.5 ± 0.2) of Si/SiO_2_ samples with composite films of albumin (green), cellulose (red), and acrylic polymer (blue) mixed with 0.45 wt.% nanotubes. The first measurement for each composition (day 0) is performed with fresh samples, samples were stored in 1 mM KCl solution for 1 week, and then, before each following experiment (day 7 and day 14, respectively). Arrows mark the propagation of the behavior for sequential measurements. Dashed lines indicate values for unmodified albumin, cellulose, and Si/SiO_2_.

Figure 1 shows typical examples of the stability measurements of the ζ-potential for the different SWCNT-polymer composites. Each composite is represented by its own color, i.e., albumin is shown in green, cellulose in red, and acrylic polymer in blue. The three dotted lines for each color represent the three cycles of stability measurements starting on day 0 (initial measurement of the virgin sample), day 7, and day 14, respectively. For comparison, we added the literature values of the ζ-potential of SiO_2_ (−(20 ± 1) mV), albumin (−(6 ± 0.5) mV), and cellulose (−(37 ± 1) mV) at pH 5.5 [47,48]. Unfortunately no values for the ζ-potential of our specific acrylic (−(21 ± 1) mV) are available.

Generally, all compositions show the similar tendencies, which are clear indications for the degradation of the films:

- In all cases, the initial ζ-potential of the as prepared sample is similar to the value expected for the polymer.
- Due to the exposure to the electrolyte, the ζ-potential changes significantly in time. The rate of the change is different for the different polymers.
- In all cases, the potential shifts towards the ζ-potential of the Si/SiO_2_ substrate. However, only in the case of acrylate, the potential gets very close to that of the substrate.

**Figure 2:**
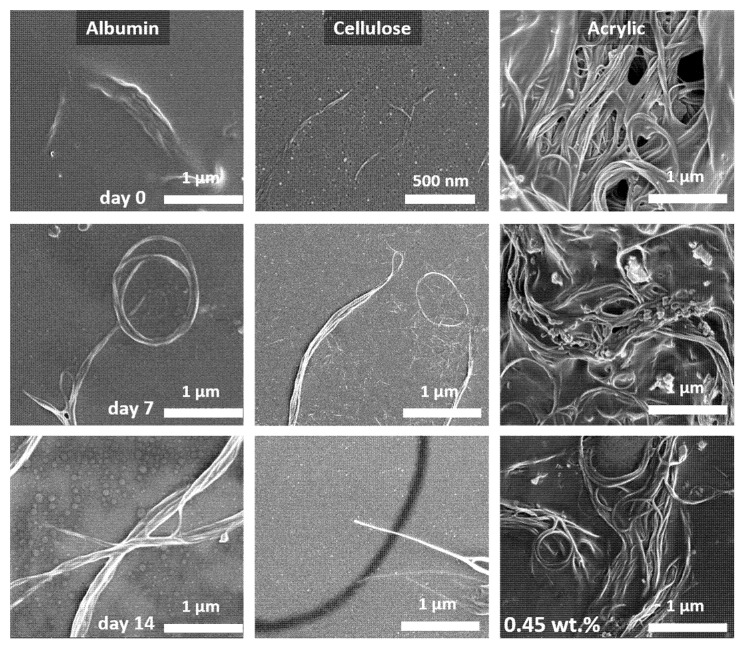
Scanning electron micrographs of Si/SiO_2_ samples with composites of albumin (first column), carboxymethylcellulose (second column), and acrylic polymer (third column) mixed with 0.45 wt.% nanotubes. The first measurement for each composition (day 0—first row) is performed with fresh samples, and the samples were stored in 1 mM KCl solution for 1 week before each following experiment (day 7 and day 14—second and third rows, respectively). Figures illustrate (i) behavior of single-walled carbon nanotubes (SWCNT) distribution (individual NT in case of albumin and cellulose and clustering in case of acrylic polymer) and (ii) visual effect of the substance degradation and development of buried single nanotubes depending on the material. Albumin and cellulose—strong degradation, acrylic—no visually observed degradation.

Nevertheless, let us discuss behavior of each material in detail:

(i) Albumin samples change their surface potential during the “day 0” measurement from −(8 ± 0.5) to −(5 ± 0.5) mV within the first hour of the experiment. This value nicely correlates with the ζ-potential of unmodified albumin −(6 ± 0.5) mV [47]. We assume that this is the case due to (i) the low concentration of nanotubes and (ii) nanotubes are mostly immersed inside the film (see Figure 2 day 0). Such strong and intense change of the surface charge is peculiar for each material during the first active stability test caused by the streaming electrolyte, which has quite significant influence on the unstable interface. We assume that this is caused by removal of the excess of silicon particles appeared after the wafer cutting (see Material part) and release of single carbon nanotubes attached to the surface via weak Van der Waals force combined with degradation of the composite itself. After the first hour, the slope changes and ζ-potential begins to decrease linearly at a rate of about 0.12 mV/h. This corresponds to the albumin biodegradation process. The degradation naturally continues during the first passive stability test in the electrolyte. As a result, the second stability test “day 7” starts at around −20 mV, nevertheless, it does not mean that albumin composite is completely dissolved during the first week (Figure 2 day 7 for albumin supports this). The surface potential rapidly restores back to less negative values. The dependence is quite noisy and stabilizes to a smother line only by the end of the experiment. This continues up to −15 mV at a 6 h’ measurement point. Samples are removed from the device and immersed in the electrolyte again. “Day 14” measurement starts at about the same point where the previous measurement was finished −(16 ± 0.5) mV. This time, the line is very smooth and the surface potential values just slightly varies around −15 mV level. It means that the major dissolving process of the composite has already occurred and just some detachment of nanotubes together with ionic exchange between surface and electrolyte take place. Albumin has the least negative surface potential value (≈−24 mV) at pH 7 (see Figure S5), making it surface, among others, the most favorable for certain types of cell (e.g., neurons or fibroblasts), without considering additional surface modification.
(ii) Cellulose samples change their surface potential during the “day 0” measurement from −(41 ± 1) to −(35 ± 1) mV. Similar to albumin samples, this agrees with literature value of unmodified cellulose −(37 ± 1) mV [48]. The first stability test results are very noisy and unstable. This means that a significant change occurs in the composite layer. Most likely, quite large areas of the materials are detached and removed with the electrolyte flow. The second stability test looks much smoother. However, a surface potential is still changing by about 8 mV within 6 h’ measurement. The “day 14” measurement is very stable and linear with a surface potential of −(31 ± 1) mV. This shows that in comparison to albumin, where a major degradation occurred already by the end of the first week, cellulose-based composite gradually dissolves within 2 weeks. Cellulose has the most negative surface potential value (≈−38 mV) at pH 7 (see Figure S5), making its surface, among others, the least favorable for certain types of cells (e.g., neurons or fibroblasts). This is further confirmed during the cell cultivation experiment.
(iii) Similar to cellulose, acrylic polymer composite changes its ζ-potential from more negative to less negative values (towards unmodified Si/SiO_2_). However, in comparison to cellulose, acrylic polymer changes its surface potential mostly within the first active stability test, i.e., from −(32 ± 0.5) to −(24 ± 0.5) mV. “Day 7” experiment starts with similar values, meaning that during passive stability, no surface changes occurred. The ζ-potential changed to −(20 ± 0.5) mV by the end of the second active stability test. It stabilizes at −(21 ± 0.5) mV and does not change during the second passive stability test and the “Day 14” test. SEM micrographs support this stable behavior showing that acrylic polymer composite does not change its morphology (see Figure 2).

### 3.2. Scanning Electron Microscopy Imaging

The scanning electron microscopy (SEM) images partially confirm the observations of the ζ-potential measurements discussed above. For the composites based on albumin and cellulose, an aging of the samples in the electrolyte is clearly visible. The morphology of the as-fabricated albumin and cellulose samples (see Figure 2 “day 0”) and those exposed to 1 mM KCl electrolyte over 7 days (Figure 2 “day 7”) clearly evolves, the films dissolve promoted by the electrolyte. Both composites degrade and start to expose buried nanotubes. This can nicely be observed for albumin. Initially only the sticking out top part of the nanotubes is exposed (image of “day 0”), however, significantly larger parts are visible after 7 days. After 14 days, nanotubes are even completely exposed (see Figure 2 “day 14”). A similar behavior is observed for cellulose samples. The significant part of cellulose together with smaller single nanotubes is washed away by day 14, whereas big, joined nanotubes are still present. They are the main electrical conductors in the leftover composite.

In contrast, the morphology of the acrylic-based composition does not show any visible changes. This can be explained by the good acrylic polymer stability in the electrolyte. However, this seems to contradict our observation of the degradation of the ζ-potential.

Nevertheless, considering an inhomogeneous three-dimensional distribution of nanotubes in acrylic (shown in Figure 2), we can assume that during the first stability test of acrylic samples (see Figure 1) a washing out of contaminants takes place. Moreover, surface potential measurement could give deviating results due to uneven surface.

### 3.3. Electrical Measurements

**Figure 3:**
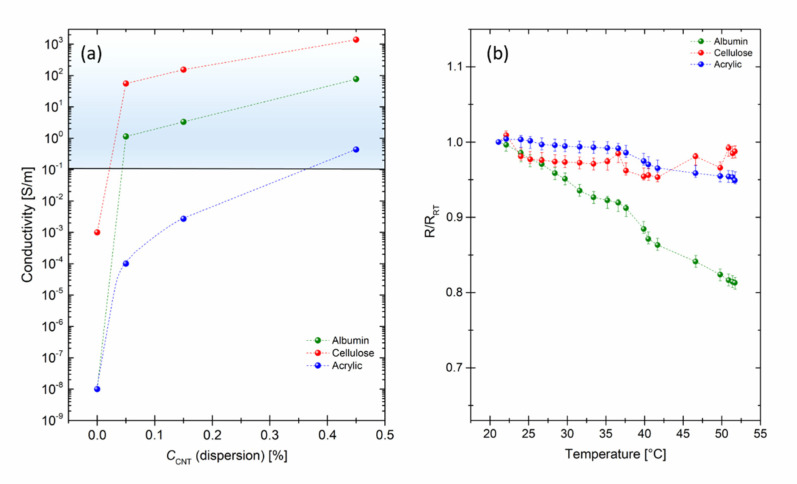
(**a**) Conductivity of albumin (green), cellulose (red) and acrylic polymer (blue)-based composites mixed with 0.05, 0.15 and 0.45 wt.% nanotubes, the blue shaded area indicates the regime of interest for medical and bio applications [3]. (**b**) Normalized resistance (R/R_RT_) as a function of temperature for albumin (green), cellulose (red) and acrylic polymer (blue)-based composites mixed with 0.45 wt.% nanotubes. Normalized values are obtained via dividing every measurement value on the first initial value (R_RT_).

The resistive measurements indicate the large potential of the composites. They demonstrate:

- The large conductivity regime of more than 6 orders of magnitude which can simply be covered by adding up to 0.45 wt.% (see Figure 3a) and at the same time,
- A very small temperature dependence of the conductivity in the relevant temperature regime (see Figure 3b).

All three base materials are intrinsically dielectrics, i.e., isolators. The conductivity of cellulose without nanotubes is about 10^−3^ S/m, the conductivity of albumin and acrylic polymer is even smaller, i.e., ≈10^−8^ S/m, whereas, an addition of 0.05 wt.% nanotubes leads to a significant rise of the conductivity for all composites. With larger amount of nanotubes (from 0.05% to 0.45 wt.% nanotubes), all composites show a further linear increase in the conductivity. The acrylic polymer-based component has the smallest conductivity among the investigated materials. Even though, the conductivity rapidly rises from 10^−4^ S/m for 0.05 wt.% nanotubes samples to 0.4 S/m for 0.45 wt.% nanotubes samples, these values are way too small for appropriate biological application. The albumin-based composites show better results, the conductivity changes from 1.1 S/m for 0.05 wt.% nanotubes samples to around 75 S/m for 0.45 wt.% nanotubes samples. Considering the linear change of the dependence, both materials can be potentially used for biological application at about 2 wt.% nanotubes and higher, however, this amount is quite large, especially considering the rapid degradation of albumin and as a result ejection of nanotubes in a living body. The cellulose-based composite shows the best performance regarding the conductivity. It ranges from 50 S/m for 0.05 wt.% nanotubes samples to 1300 S/m for 0.45 wt.% nanotubes samples. Next, we performed a temperature stability test for the samples mixed with 0.45 wt.% nanotubes in the range from room temperature (20 °C) to 51.7 °C (see Figure 3b). We performed 20 measurements for each sample within the selected range. Albumin turned out to be the least persistent composite. After heating up, its resistance decreased down to 80% of the initial resistance measured at room temperature. Still, if we take a point of 36.6 °C, considering a possible application within a human body, the resistance is at about 92% of the initial index, which is already a rather good result. During the measurement of cellulose and acrylic-based composites, the resistance did not fall below 95% of the initial resistance. Both composites show outstanding stability within the measurement range.

**Figure 4:**
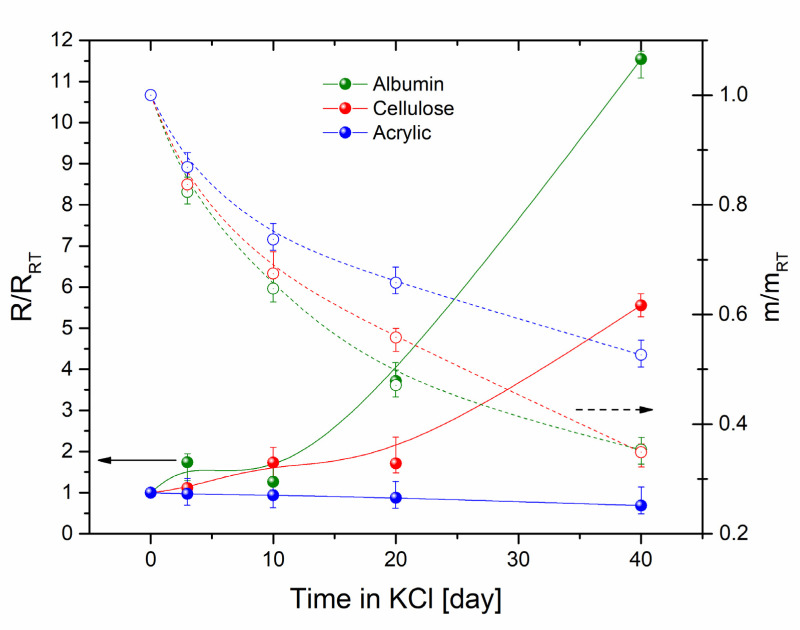
Stability test of albumin (green), cellulose (red) and acrylic polymer (blue)-based composites mixed with 0.45 wt.% nanotubes in 0.1 mM KCl electrolyte showing changes of the normalized resistance (R/R_RT_) (left scale, straight lines) and the normalized mass (m/m_RT_) of deposited composites (right scale, dashed lines). R_RT_ and m_RT_ are initial resistance and mass values used for normalization, respectively.

Finally, we investigated the stability of the samples mixed with 0.45 wt.% nanotubes kept in 0.1 mM KCl solution in order to correlate the results with our ζ-potential stability tests. Samples were measured after the preparation, then immersed into electrolyte and measured again at day 3, 10, 20, and 40 (see Figure 4). Simultaneously with the resistive measurements, the mass change of the composites was investigated. In conformity with SEM data (see Figure 2), acrylic-based composite has the least pronounced change in conductivity and mass. The resistance in this case has almost not changed, whereas the resistance of cellulose and albumin composites increased 6 and 12 times by day 40, respectively. Considering that the mass of cellulose and albumin-based composites decreased down to 35% of the initial values, we assume that albumin loses more SWCNT from its composition than cellulose at the same time. Therefore, it results in lower conductivity of albumin, which in the combination with rapid outburst of nanotube during the degradation of the material in the electrolyte; this represents the drawback of albumin in comparison to cellulose-based composite.

### 3.4. Fibroblast Cell Growth on Composites

In the last step, we analyze the growth of fibroblast cells on different conductive composites. For comparison, Si/SiO_2_ samples (only chemically cleaned) are added as a reference.

**Figure 5:**
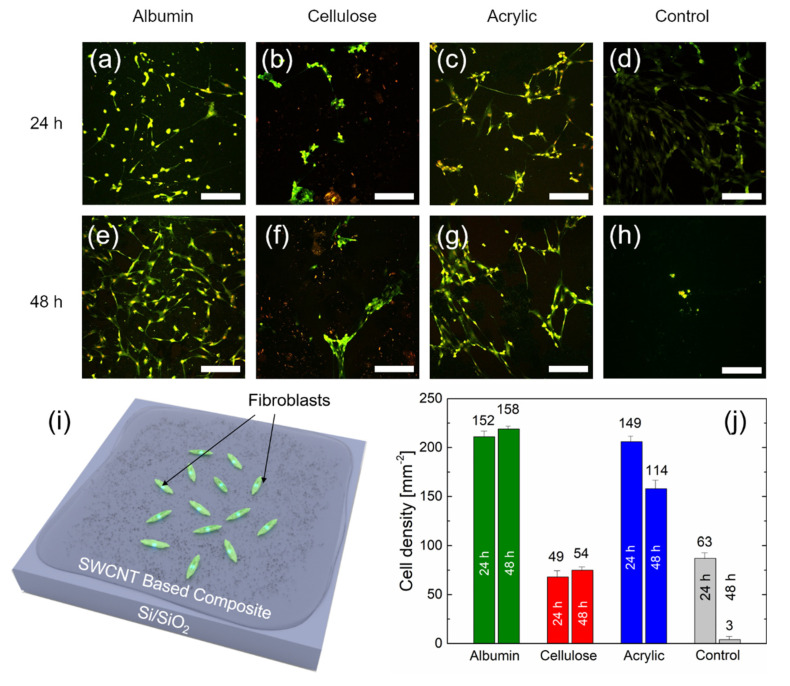
(**a**–**h**) Examples of fluorescence microscope images (850 μm × 850 μm) of fibroblast cell cultures on Si/SiO_2_ substrates with different composites after 24 and 48 h in vitro: albumin (**a**,**e**), cellulose (**b**,**f**), and acrylic polymer (**c**,**g**)-based composites mixed with 0.45 wt.% SWCNT and control samples (i.e., Si/SiO_2_ substrates without composites) (**d**,**h**). The scale bar for (**a**–**h**) is 200 µm. (**i**) Schematic of fibroblasts immobilized on a Si/SiO_2_ sample with SWCNT-based composite. The values on the bars in (**j**) represent the total number of live cells averaged over several areas of size 850 μm × 850 μm (0.7225 mm^2^). The results from three images on three different samples for each type of the composite and day in vitro (DIV) were always averaged for the statistics, *n* = 9.

Figure 5 shows the density of living fibroblast cells obtained on SiO_2_ substrate with different SWCNT-based composites mixed with 0.45 wt.% nanotubes, and the reference samples (Si/SiO_2_ without composites) after 24 and 48 h in vitro (schematic image is given in Figure 5i). Figure 5a–h shows examples of fluorescence images obtained after live cell staining (see Materials and Methods), whereas in Figure 5j, the statistics derived from these images are presented. The main results of this experiment are as follows:

- Although cell cultures on albumin and acrylic polymer show live cell densities significantly higher than the reference samples, the fibroblast live density on cellulose is only insignificantly lower;
- The investigated SWCNT results in the better proliferation of cells within 48 h in vitro in comparison to reference samples.

Let us discuss these results in detail:

Among other biomedical applications and due to their electrical conductivity and elastic properties, SWCNT-based nanocomposites can be used in the form of thin films, e.g., as deformation sensors applied to a human skin [53], for a cell proliferation [54], a tissue regeneration [55], or substrates for cells stimulation devices [56]. Therefore, in order to demonstrate the potential of our composite layers for thin films biological applications, we immobilized fibroblasts on different composites mixed with 0.45 wt.% nanotubes. The highest concentration of SWCNT was chosen since we assume that nanotubes in the biocomposite initially reduce the cell growth density. Therefore, if the cell culture can survive the highest concentration, then the cell growth density would even increase with decreasing SWCNT concentration in the composite. The fluorescence images (Figure 5a−h) show exemplary images of living fibroblasts cultured directly on the SWCNT-based nanocomposites within 24 and 48 h. For the 24 h reference (Figure 5d) samples, cell densities of ~90 mm^−2^ are obtained, which is comparable to standard values for fibroblast-coated substrates [57]. For 24 h albumin and acrylic polymer-based samples (Figure 5a,c), the density of cells increases strongly (~210 mm^−2^) in comparison to the reference. The pronounced difference between these two composites is that in case of albumin-based samples, cells are distributed more evenly than in case of acrylic-based samples, where cells tend to form clusters. The cell density is generally lower for cellulose-based samples (Figure 5b) in comparison to the reference (~70 mm^−2^). Moreover, fibroblasts in this case similar to acrylic polymer-based samples forms separate clusters.

For 24 and 48 h in vitro study, there is no much difference between albumin, cellulose, and acrylic polymer-based samples. The only observed distinction is a reduction in the cell density for acrylic-based samples to ~160 mm^−2^. However, the major difference is that in case of 48 h’ reference samples, the cell density drops dramatically down to ~40 mm^−2^. This suggests that almost no cells survive more than 48 h in vitro on the reference samples. In general, the ability of biocomposites to improve the cell adhesion is not novel [58,59]. However, our research is very interesting since it suggests that SWCNT-based composites on SiO_2_ not only increase the fibroblasts cell density (in case of albumin and acrylic polymer-based samples) but also create conditions for a longer life of cells on the samples’ surface. This underlines the significance of this work, because the SWCNT-based composites do not only seem to enable a tailoring of surface properties in terms of increasing the electrical conductivity by several orders of magnitude but also provide the ability to use these properties in bioelectronics and biomedicine applications. Moreover, it is of a great importance that SWCNT-based materials have a potential translatability into clinical applications [60].

## 4. Conclusions

In the present paper, we describe a way to engineer biocompatible conductive biocompatible composites based on bovine serum albumin, carboxymethylcellulose, and acrylic polymer with 0.05, 0.15, and 0.45 wt.% of single-walled carbon nanotubes. By varying the ratio of the SWCNT, we can tune the overall conductivity from 10^−4^ to 10^3^ S/m. We observe various velocities of biodegradation and stabilization of the surface properties via ζ-potential measurement. We demonstrate significant differences in surface charge for the different composites, as well as altered surface potential within the pH range from 5.5 to 9.3 in the 0.1 mM KCl electrolyte. SEM imaging represents differences in the distribution of SWCNTs in the composite, ranging from scattered single nanotubes in case of albumin samples to clustered “spaghetti” in case of acrylic polymer samples. Fluorescence microscopy demonstrates the biocompatibility of these composites utilizing fibroblasts cell cultures. The cell density on albumin and acrylic polymer samples is ~2.5 times larger than that of the reference samples. Moreover, the nanocomposite coating influences the lifespan of cells on the surface. The concentration of cells on the composite remained unchanged for 48 h in vitro, while on the reference samples, almost no cells survived.

In conclusion, their material properties like (i) the surface potential and its’ stability, (ii) biodegradation, (iii) conductivity, and (iv) biocompatibility make SWCNT-based composites are interesting for many biomedical applications. Furthermore, all of the above should be correlated with safety related issues. This is why it is of importance to investigate the release rate of the nanotubes (i.e., biodegradation time). Our preliminary experiments showed that they could be good candidates for the engineering of strain gauges, pressure sensors, electrodes, or elastomers leading to a broad application range including skin electronics, tissue regeneration, and cell stimulation.

## Supplementary Materials

The following are available online at https://www.mdpi.com/2079-4991/10/12/2492/s1. Figure S1: Schematic of the major component of the streaming current measurements setup; Figure S2: High magnification fluorescence microscope image of fibroblasts; Figure S3: Scanning electron micrograph of single-walled carbon nanotubes; Figure S4: Scanning electron micrograph of Si/SiO_2_ sample with bovine serum albumin; Figure S5: ζ-potential values of Si/SiO_2_ samples with composite films as a function of pH value for 1 mM KCl electrolyte; and Table S1: Components and composition of the used water dispersions.
