# Supplementary material for: Biocompatible SWCNT Conductive Composites for Biomedical Applications
Source: Nanomaterials (Basel). 2020 Dec 11;10(12):2492. doi: 10.3390/nano10122492 (PMC7763503; doi:10.3390/nano10122492)
Supplement: Supplementary file 1 [file nanomaterials-10-02492-s001.pdf]

nanomaterials

Supporting Information

Biocompatible SWCNT Conductive Composites for Biomedical Applications

Aleksandr Markov*[1], Roger Wördenweber[2], Levan Ichkitidze[1,3], Alexander Gerasimenko[1,3], Ulyana Kurilova[3], Irina Suetina[4], Marina Mezentseva[4], Andreas Offenhäusser[2] and Dmitry Telyshev[1,3]

[1] Institute for Bionic Technologies and Engineering, I. M. Sechenov First Moscow State Medical University, 119991, Moscow, Russian Federation; ichkitidze@bms.zone (L.I.); gerasimekno@bms.zone (A.G.); telyshev@bms.zone (D.T.)
[2] Institute of Biological Information Processing, Bioelectronics (IBI-3), Research Center Jülich, Jülich 52425, Germany; r.woerdenweber@fz-juelich.de (R.W.); a.offenhaeusser@fz-juelich.de (A.O.)
[3] Institute of Biomedical Systems, National Research University of Electronic Technology, Zelenograd, 124498, Moscow, Russian Federation; kurilova@bms.zone (U.K.)
[4] Ivanovsky Institute of Virology, National Research Center for Epidemiology and Microbiology named after the honorary academician N.F. Gamaleya, 123098, Moscow, Russian; ikas@inbox.ru (I.S.); marmez@mail.ru (M.M.)
* Correspondence: markov@bms.zone (A.M.)

Supplementary figures

S-1

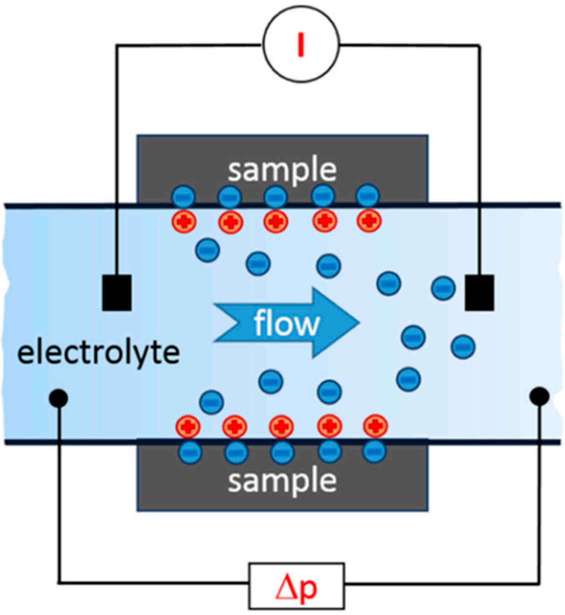

Figure S1. Schematic of the major component of the streaming current measurements setup consisting of a pair of identical planar substrates placed in a clamping cell with the surfaces to be analyzed facing each other and forming a microfluidic channel, as well as electrodes and sensors to determine the electric current as a function of the flow of the electrolyte.

S-2

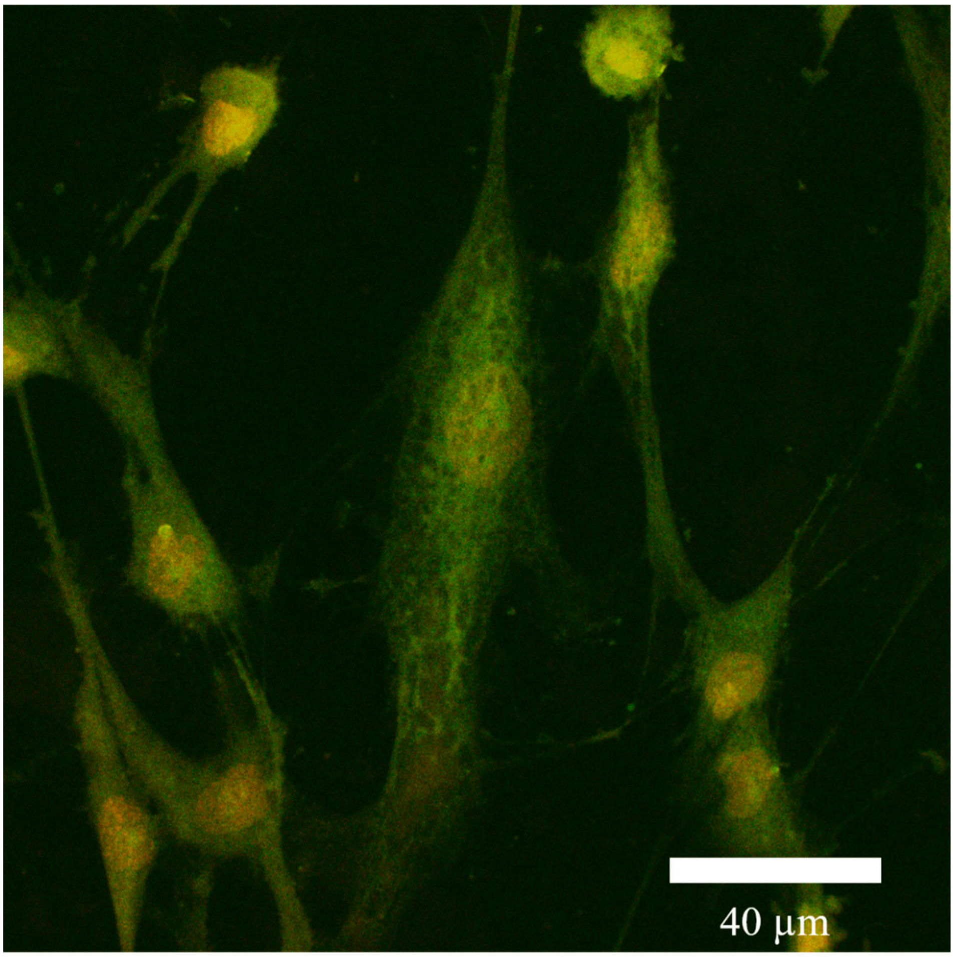

Figure S2. High magnification fluorescence microscope image of the fibroblast culture after 48 hours in vitro on bovine serum albumin based composites mixed with 0.45 wt.% SWCNT.

S-3

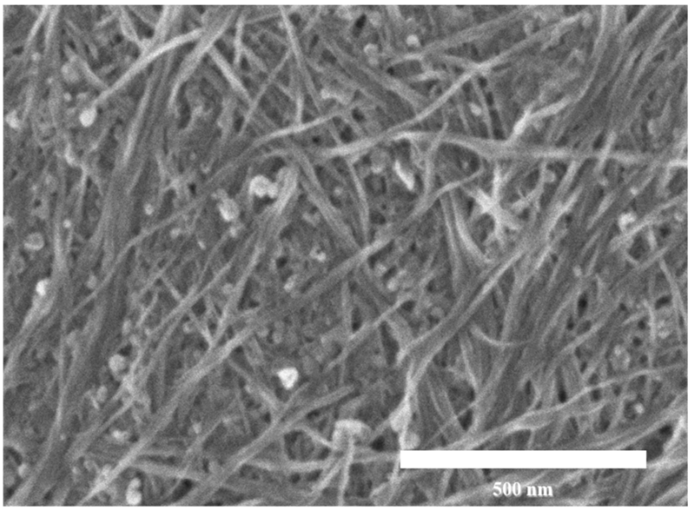

Figure S3. Scanning electron micrograph of single walled carbon nanotubes.

S-4

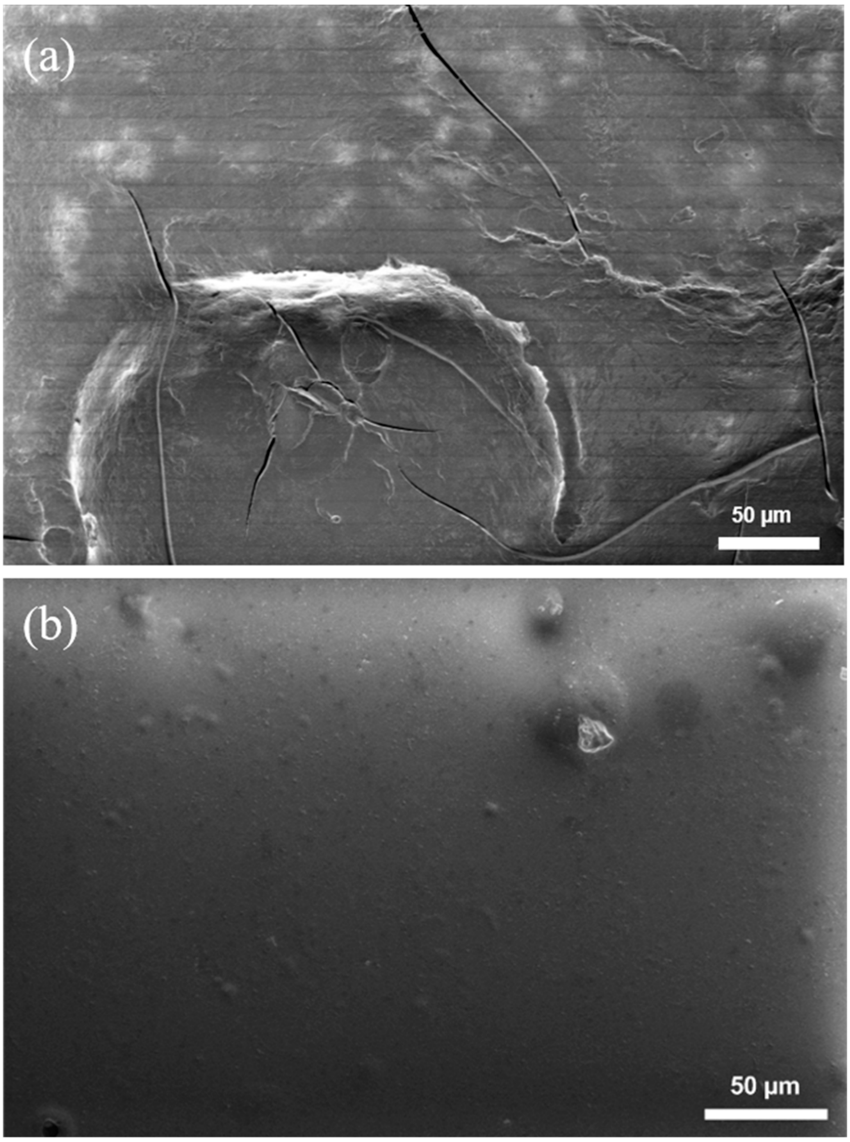

Figure S4. Scanning electron micrograph of Si/SiO_2 sample with bovine serum albumin (without nanotubes) with cracks (a) and without cracks (b).

S-5

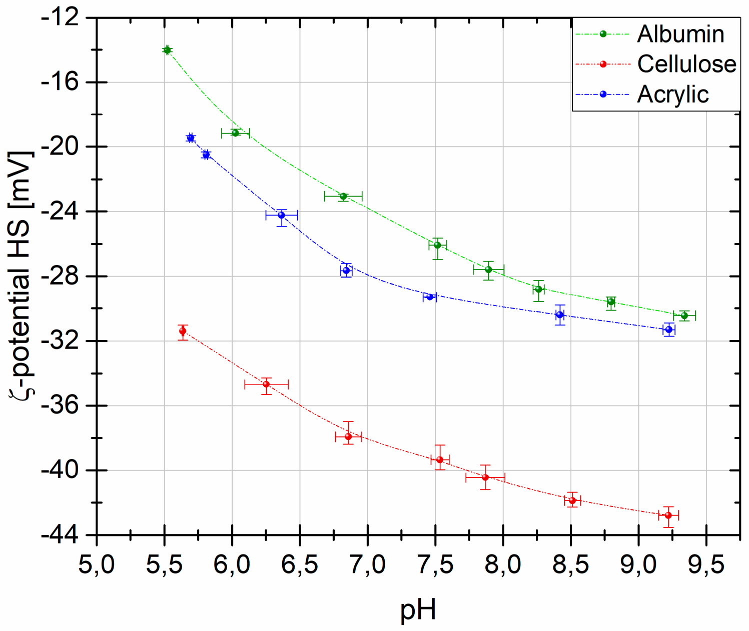

Figure S5. ζ-potential values of Si/SiO2 samples with composite films of albumin (green), cellulose (red) and acrylic polymer (blue) mixed with 0.45% wt nanotubes directly after third stability test (day 14) as a function of pH value for 1mM KCl electrolyte.

Supplementary tables

Table S1. Components and composition of the used water dispersions based on bovine serum albumin (BSA), carboxymethylcellulose (CMC) and acrylic

**Water dispersions**
25 wt. % BSA / 0.45 wt. % SWCNT
25 wt. % BSA / 0.15 wt. % SWCNT
25 wt. % BSA / 0.05 wt. % SWCNT
3 wt. % CMC / 0.45 wt. % SWCNT
3 wt. % CMC / 0.15 wt. % SWCNT
3 wt. % CMC / 0.05 wt. % SWCNT
20 wt. % AP / 0.45 wt. % SWCNT
20 wt. % AP / 0.15 wt. % SWCNT
20 wt. % AP / 0.05 wt. % SWCNT

Publisher's Note: MDPI stays neutral with regard to jurisdictional claims in published maps and institutional affiliations.

© 2020 by the authors. Submitted for possible open access publication under the terms and conditions of the Creative Commons Attribution (CC BY) license (http://creativecommons.org/licenses/by/4.0/).
